# A Soybean Deletion Mutant That Moderates the Repression of Flowering by Cool Temperatures

**DOI:** 10.3389/fpls.2020.00429

**Published:** 2020-04-15

**Authors:** Jingyu Zhang, Meilan Xu, Maria Stefanie Dwiyanti, Satoshi Watanabe, Tetsuya Yamada, Yoshihiro Hase, Akira Kanazawa, Takashi Sayama, Masao Ishimoto, Baohui Liu, Jun Abe

**Affiliations:** ^1^Research Faculty of Agriculture, Hokkaido University, Sapporo, Japan; ^2^Key Laboratory of Soybean Molecular Design Breeding, Northeast Institute of Geography and Agroecology, Chinese Academy of Sciences, Harbin, China; ^3^Faculty of Agriculture, Saga University, Saga, Japan; ^4^Takasaki Advanced Radiation Research Institute, National Institutes for Quantum and Radiological Science and Technology, Takasaki, Japan; ^5^Western Region Agricultural Research Center, National Agriculture and Food Research Organization, Zentuji, Japan; ^6^Institute of Crop Science, National Agriculture and Food Research Organization, Tsukuba, Japan; ^7^School of Life Sciences, Guangzhou University, Guangzhou, China

**Keywords:** ambient temperature, *CONSTANS*, *E1*, flowering, *Glycine max*, mutant, soybean

## Abstract

Ambient growing temperature and photoperiod are major environmental stimuli that summer annual crops use to adjust their reproductive phenology so as to maximize yield. Variation in flowering time among soybean (*Glycine max*) cultivars results mainly from allelic diversity at loci that control photoperiod sensitivity and *FLOWERING LOCUS T* (*FT*) orthologs. However, variation in the thermal regulation of flowering and its underlying mechanisms are poorly understood. In this study, we identified a novel mutant (ef1) that confers altered thermal regulation of flowering in response to cool ambient temperatures. Mapping analysis with simple sequence repeat (SSR) markers located the mutation in the upper part of chromosome 19, where no QTL for flowering has been previously reported. Fine-mapping and re-sequencing revealed that the mutation was caused by deletion of a 214 kbp genomic region that contains 11 annotated genes, including *CONSTANS-LIKE 2b* (*COL2b*), a soybean ortholog of *Arabidopsis CONSTANS*. Comparison of flowering times under different photo-thermal conditions revealed that early flowering in the mutant lines was most distinct under cool ambient temperatures. The expression of two *FT* orthologs, *FT2a* and *FT5a*, was dramatically downregulated by cool temperature, but the magnitude of the downregulation was lower in the mutant lines. Cool temperatures upregulated *COL2b* expression or delayed peak expression, particularly at the fourth trifoliate-leaf stage. Intriguingly, they also upregulated *E1*, a soybean-specific repressor of *FT* orthologs. Our results suggest that the *ef1* mutation is involved in thermal regulation of flowering in response to cool ambient temperature, and the lack of *COL2b* in the mutant likely alleviates the repression of flowering by cool temperature. The *ef1* mutant can be used as a novel gene resource in breeding soybean cultivars adapted to cool climate and in research to improve our understanding of thermal regulation of flowering in soybean.

## Introduction

Crops have been developed to produce greater yields by adjusting their reproductive phenology in response to external signals such as photoperiod and ambient temperature. Soybean (*Glycine max*) is a major legume crop cultivated worldwide for human food, animal feed, and oil production for edible and industrial uses. Its wide adaptability has been supported by the development of diverse cultivars with different photoperiod sensitivities, although cultivation of individual cultivars may be restricted to relatively narrow ranges of latitudes. Variations in flowering time among soybean cultivars have been attributed mainly to the genetic variation at loci involved in photoperiod responses, such as the loci for the phytochrome A (*PHYA*) – *E1* and *GIGANTEA* – *CONSTANS* modules (Cao et al., 2017).

The maturity gene *E1* is a repressor of flowering under long days (Xia et al., 2012). It encodes a legume-specific putative transcription factor that contains a bipartite nuclear localization signal and a region distantly related to the B3 domain; overexpression of *E1* strongly represses *FT2a* and *FT5a*, the soybean orthologs of *Arabidopsis FLOWERING LOCUS T* (*FT*) (Kong et al., 2010), and inhibits floral initiation (Xia et al., 2012). The expression of *E1* and its homologs, the *E1*-*like* genes (*E1La* and *E1Lb*), is controlled by the maturity genes *E3* and *E4*, which encode two PHYA proteins, PHYA3 and PHYA2, respectively; the expression of *E1* and *E1L* genes is induced under long days but is repressed under short days and in the *e3*/*e4* double recessive homozygote (Xia et al., 2012; Xu et al., 2015). The *J* gene, which is responsible for long juvenility (Ray et al., 1995), encodes an ortholog of *Arabidopsis EARLY FLOWERING 3* (*ELF3*) (Lu et al., 2017; Yue et al., 2017). Under short-day conditions, the J protein directly binds to the promoter of *E1*, inhibiting its expression; in plants that are homozygous for the dysfunctional *j* allele, *E1* expression is induced even under short-day conditions, and strictly inhibits flowering (Lu et al., 2017). *E2*, another important maturity gene, is an ortholog of *Arabidopsis GIGANTEA* (*GI*); it inhibits flowering under long days, mainly by suppressing *FT2a* expression (Watanabe et al., 2011). The recessive *e2* allele is predominant in early-maturing (Wang et al., 2016) and photoperiod-insensitive cultivars (Xu et al., 2013).

*FT2a* and *FT5a* contain rich nucleotide polymorphisms in the promoter and coding regions, some of which are associated with different flowering times among cultivars (Takeshima et al., 2016; Zhao et al., 2016; Jiang et al., 2019; Ogiso-Tanaka et al., 2019; Sun et al., 2019). Genetic variation in flowering time among soybean cultivars is therefore attributable to the allelic diversity at loci involved in the *PHYA-E1* module (*E1*, *E3*, and *E4*), as well as *E2* and two floral integrators, *FT2a* and *FT5a* (Cao et al., 2017). Diverse allelic combinations at these loci have enabled soybean to adapt to a wide range of latitudes.

Ambient growth temperature also has a large impact on reproductive development in soybean (Hadley et al., 1984; Upadhyay et al., 1994; Cober et al., 2001; Thomas et al., 2010; Kumagai and Sameshima, 2014; Mao et al., 2017; Sun et al., 2019). The thermal response of flowering in soybean varies in response to both photoperiod and maturity genotype. At photoperiods below the critical daylength, increasing temperatures accelerate flowering up to an optimum temperature (∼30°C), but further temperature increases delay flowering (Hadley et al., 1984; Upadhyay et al., 1994; Cober et al., 2001; Thomas et al., 2010). Cober et al. (2001) found different responses of flowering to high temperature between photoperiod-sensitive and -insensitive genotypes with a genetic background of Harosoy, an early-maturing cultivar; flowering of photoperiod-sensitive genotypes, particularly in a photoperiod of 20 h, was noticeably delayed at 28°C relative to 18°C, whereas that of insensitive genotypes was not delayed. Furthermore, Sun et al. (2019) found a delay of flowering at 32°C in a photoperiod-insensitive line with a late-flowering trait introduced from a Thai cultivar, a unique characteristic that has not been reported in ordinary photoperiod-insensitive lines. However, the genetic variation and the underlying molecular mechanism of its effect on thermal regulation of flowering, particularly in response to cool temperatures, are not yet understood in soybean.

In this study, we characterized flowering behaviors of an early-flowering mutation selected from the mutant population previously induced by ion-beam radiation (Arase et al., 2011) under different photo-thermal conditions, and identified the DNA polymorphism responsible for the mutation by mapping and re-sequencing. The mutation is involved in the thermal regulation of the response of flowering to cool temperatures but not in photoperiod sensitivity, and was caused by deletion of a 214 kbp region in the upper part of chromosome 19, where no QTL for flowering has been reported. The deletion contained *CONSTANS-LIKE 2b* (*COL2b*), which is a soybean ortholog of *Arabidopsis CONSTANS* (*CO*). Expression profiling further revealed that although cool temperatures downregulated *FT2a* and *FT5a* expression, the magnitude of the downregulation was lower in the mutant. Our data therefore suggest a possible involvement of a *CO-like* gene in the thermal regulation of flowering, particularly in response to cool weather.

## Materials and Methods

### Plant Materials

We selected a new early-flowering mutant (N2-ef1) from a mutant population of Nourin No. 2 (N2) generated by ion-beam irradiation (Arase et al., 2011), from which several mutants with altered flowering have been selected and characterized (Mikuriya et al., 2017). We crossed N2-ef1 with the early-flowering cultivar “Tokachi Nagaha” (TN) in place of N2 to facilitate mapping of the mutant with simple sequence repeat (SSR) markers. We evaluated segregation of the flowering time in the F_2_ population at the Institute of Crop Science, Tsukuba, Japan (36.03°N, 140.10°E), but performed the subsequent genetic analyses at Hokkaido University, Sapporo, Japan (43.07°N, 141.34°E). Single F_3_ seeds from each of 119 F_2_ plants, together with seeds of the parents (N2-ef1 and TN), were sown in paper pots (Paperpots No. 2, Nippon Beet Sugar Manufacturing Co., Tokyo, Japan) on 14 June 2013, and were then transplanted 10 days later at the experimental farm of Hokkaido University. A piece of undeveloped young trifoliate leaves was sampled from each F_3_ plants, and the individual flowering times (number of days after sowing; DAS) were recorded. The segregation of flowering time was examined in 13 F_3_ plants that were selected on the basis of the genotype at a tagging marker (Sat_405, detected from the association test described later in the Methods) for the mutant gene in N2-ef1 in 2014 (12 June sowing date) and in 25 F_4_ plants from three F_3_ families in 2015 (5 June sowing date). The numbers of plants tested in the progeny were 15–56 plants in the F_3_ plants, and 10–186 plants in F_4_ plants.

Genetic analysis and fine-mapping of the mutant gene were carried out in the progeny of a heterozygous F_4_ plant (#46-16) from 2016 to 2019 (28 May to 6 June sowing dates). We tested 15 plants in the progeny test. We developed near-isogenic lines (NILs) for the mutant and wild-type alleles in the progeny from two heterozygous F_5_ plants (#46-14-32 and #46-14-69). We treated the offspring of an early-flowering F_6_ plant that was homozygous for the allele from N2-ef1 at the tagging marker as a NIL for ef1 and the offspring of a late-flowering plant homozygous for the allele from TN as a NIL for the wild-type.

### Evaluation of Photo-Thermal Responses

Two sets of NILs were grown under different combinations of photoperiod and temperature. We examined combinations of daylengths of 14, 16, and 18 h at a constant air temperature of 25°C and a daylength of 16 h at constant temperatures of 18, 25, and 32°C. Three photoperiod conditions were set in a greenhouse at Hokkaido University in the winter season of 2018–2019. Air temperatures in the greenhouse were adjusted to 25°C, with fluctuations from a minimum of 22°C to a maximum of 28°C; lighting was supplied using high intensity discharge lamps (HONDA-T, Panasonic Co, Osaka, Japan) with an average photosynthetically active photon flux density of 120 μmol m^–2^ s^–1^ and a red-to-far red (R:FR) ratio of 4.5 at 1 m below the light source. Three temperature conditions were set in the growth chambers (KG50, Koito Electric Industries, Ltd., Yokohama, Japan); lighting was supplied for 16 h using a combination of fluorescent and incandescent lamps with an average photosynthetically active photon flux density of 150 μmol m^–2^ s^–1^ and an R:FR ratio of 7.0. The NILs were also grown in the greenhouse and outdoors following different sowing dates (11, 21, and 31 May) in 2019. Four pots for each line, with four plants per pot, were maintained in the greenhouse during the first 10 DAS, and then half of the pots were moved outdoors, while the other half remained in the greenhouse. Average temperatures for the 20 days after the initiation of treatment were 23.7, 22.5, and 25.9°C in the greenhouse and 18.4, 17.5, and 19.8°C in the field in the 11, 21, and 31 May sowings, respectively. Average daylengths for the first 20 days after the treatment ranged from 15 h 10 min to 15 h 20 min in the three sowing treatments (i.e., the difference was negligible). Seeds were directly sown into plastic pots (15 cm in diameter and depth), and then thinned after emergence to four plants per pot. Flowering time of eight plants (two pots) was then examined. Flowering times were recorded individually and expressed as DAS.

### DNA Extraction and Marker Analysis

Total DNA was extracted individually from leaves sampled from the parents and segregants using the modified CTAB method (Doyle and Doyle, 1990) or from seeds of heterozygous plants using the proteinase K DNA extraction method (Kamiya and Kiguchi, 2003). We analyzed polymorphisms between N2-ef1 and TN at 160 SSRs using the SSR genotyping panel developed by Sayama et al. (2011). Of these, we used 111 polymorphic SSR markers to detect significant associations between flowering time and marker genotype in 119 F_3_ plants by one-way analysis of variance (Supplementary Table S1). In the subsequent generations, we used the tagging markers and their flanking BARCSOY SSR markers (Song et al., 2010) to narrow the genomic region that harbored the mutation. Finally, we genotyped 500 seeds from heterozygous plants using two markers (BARCSOY 19_279 and 19_330). Plants derived from seeds that were recombinant in the targeted region were grown in the greenhouse during the winter of 2018, and the flowering genotypes were estimated from the segregation in the progeny in the summer of 2019. The genomic position of the mutation was determined by comparison of the estimated genotypes with the graphical genotypes constructed using the BARCSOY SSR markers. SSR marker analysis was performed as described previously (Zhao et al., 2016). The primers used in the fine-mapping are listed in Supplementary Table S2.

### Construction of Genomic Sequences Harboring the Mutation Based on Whole-Genome Resequencing Data

Raw reads of N2 and N2-ef1 were obtained by next-generation sequencing on a HiSeq XTen sequencer (Illumina, San Diego, CA, United States) and were aligned to the soybean reference genome Williams 82 (Phytozome v. 12.1/*Glycine max Wm82.a2. v1*^1^ Schmutz et al., 2010) in Bowtie 2-2.2.9 software (Langmead et al., 2009)^2^. The resulting alignment was further processed to remove duplicate reads and to correct mate and its pair information in the Picard tools^3^. Small indels were realigned in GATK v. 3.8 software^4^ (McKenna et al., 2010), in which the Unified Genotyper function, which filtered out reads with a mapped base-quality Phred score of <20, called variants (SNPs and indels). Using the reference genome and a SNP dataset for each cultivar, we reconstructed sequences of the targeted genomic regions using the FastaAlternateReferenceMaker function of GATK, and compared the sequences between N2-ef1 and N2 using the Integrative Genomics Viewer (Robinson et al., 2011)^5^. The resequencing data used in this study were submitted under BioProject accession number PRJNA600284 in NCBI.

### Sequencing of the Genomic Region Flanking a Deletion

We confirmed a deletion in the target region for the mutation in N2-ef1 by means of PCR amplification and sequencing. Two sets of primers were designed for the boundaries of the deletion, based on the re-sequenced data obtained from the deep-sequencing analyses for N2 and N2-ef1. The boundary regions were amplified by PCR with the primers F1 (5′-GAGTGGAAGATGACTAATGCAAGGT-3′) and R2 (5′-AGATGGTTTCCGGATGAAATGATTTGGG-3′) in N2-ef1, and with the primers F1 and R1 (5′-GACATTTTTGGGTATGTTTTCTTAG-3′) and F2 (5′-GTTAAGCTCATTTAAAGGATCCAAGT-3′) and R2 in N2 and TN. The amplified fragments from N2-ef1 were cloned into a pGEM-T Easy vector (Promega, Durham, NC, United States) and sequenced with a BigDye Terminator v. 3.1 Cycle Sequencing kit and an ABI PRISM 3100 Avant Genetic Analyzer (both from Applied Biosystems, Tokyo, Japan) according to the manufacturer’s instructions. We used PCR with three of the primers (F1, R1, and R2) as a deletion marker (DEL214) to determine the presence or absence of the deletion in the segregating population. The PCR products were separated by electrophoresis in 2% agarose gel, stained with ethidium bromide, and visualized under UV light.

### Expression Analyses

Expression analyses for the two *FT* orthologs, *FT2a* (Glyma.16g150700) and *FT5a* (Glyma.16g044100), and their repressors, *COL2b* (Glyma.19G039000) and *E1* (Glyma.06g207800), were performed using fully expanded new leaves of the NILs grown in the growth chambers. Leaves were sampled at Zeitgeber times (ZT) of 3, 12, and 21 h in the second and fourth trifoliate leaf stages. All samples were immediately frozen in liquid nitrogen and stored at -80°C until analysis. Total RNA was isolated from each sample using an RNeasy Plant RNA Mini Kit (Invitrogen, Waltham, MA, United States). cDNA was synthesized from total RNA (1 μg) using an oligo(dT)20 primer and random primer cocktail [non-adeoxyribonucleotide mix: pd (N)9, Takara Bio, Otsu, Japan] with M-MLV Reverse Transcriptase (Invitrogen) in a 20 μL volume, according to Khan et al. (2009). The transcript levels were determined by quantitative real-time PCR (qRT-PCR). In brief, each qRT-PCR mixture (20 μL) contained 5 μL of the cDNA synthesis reaction mixture diluted to 1/30th of its original volume, 5 μL of 1.2 μM primer premix, and 10 μL of TB Green Premix ExTaq II (TaKaRa, Kyoto, Japan). Expression levels were quantified on a CFX96 Real-Time System (Bio-Rad, Hercules, CA, United States) with PCR cycling conditions of 95°C for 3 min, followed by 40 cycles of 95°C for 10 s, 62°C for 20 s, 72°C for 20 s, and 78°C for 2 s. Values were normalized to 18S ribosomal RNA (18S rRNA). A reaction mixture without reverse transcriptase was also used as a control to confirm the absence of genomic DNA contamination. The amplification of a single DNA fragment was confirmed by a melting curve analysis and gel electrophoresis of the PCR products. Averages and standard errors of relative expression levels were calculated for three independently synthesized cDNAs. Primers used in the expression analyses are listed in Supplementary Table S3.

## Results

### Segregation Analysis and Mapping of the Early-Flowering Locus in the N2-ef1 Mutant

We previously selected and characterized mutants with altered flowering in the mutant population of Nourin No. 2 generated by ion-beam irradiation (Arase et al., 2011; Mikuriya et al., 2017). All of the mutants had yellowish leaves with reduced chlorophyll contents, and also showed promoted or delayed flowering. The mutant N2-ef1 used in this study was separately selected from the same population. It flowered ∼20 days earlier than the wild-type N2 after sowing at standard times in Sapporo (i.e., late May), but showed no other phenotypic aberration.

**FIGURE 1:**
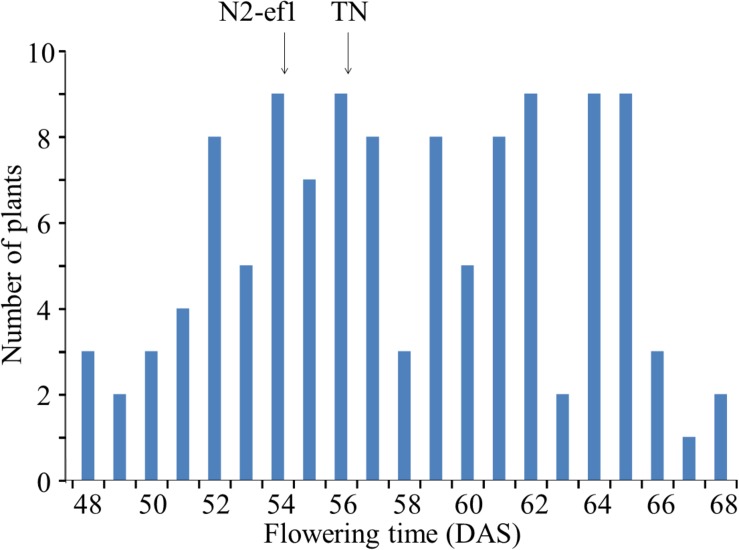
Segregation of flowering time in the F_3_ population of the cross between Nourin No. 2 early-flowering mutant 1 (N2-ef1) and “Tokachi Nagaha” (TN) in Sapporo. DAS, number of days after sowing. Arrows represent the mean flowering times of the parent accessions.

TN, which was crossed with N2-ef1 in mapping of the mutant gene using SSR markers, had the same maturity genotype as N2 at four major loci (*E1*/*e2*/*e3*/*E4*) but, as in N2-ef1, flowered ∼20 days earlier than N2 in Sapporo. Flowering time in the F_3_ population varied continuously across the flowering times of the parents (Figure 1). The parents did not differ greatly in their average flowering times: 54.4 DAS in N2-ef1 and 56.0 DAS in TN. In contrast, the F_3_ population showed a wide variation, ranging from 48 to 68 DAS, which suggests that in addition to the mutant gene, a number of unknown flowering genes that affect the difference in flowering times between N2 and TN segregated in the population.

We used one-way analysis of variance to detect significant associations between the SSR marker genotype and flowering time. Nine of the 111 polymorphic markers we tested were associated with significant differences in average flowering times between plants that were homozygous for the allele from N2-ef1 (genotype AA) and those for TN (genotype BB) (Table 1). Five markers on chromosomes (chr) 2, 9, and 11 showed that the alleles from TN promoted flowering: plants with the BB genotype flowered, on average, 2.6–3.7 days earlier than those with the AA genotype. In contrast, four markers, one each on chr4 and chr15 and two on chr19, had the opposite effect: plants with the BB genotype flowered, on average, 2.5–7.9 days later than those with the AA genotype. Because the marker Sat_405 on chr19 had the largest effect and the A allele conditioned early flowering, we focused on the genetic factor that co-segregated with Sat_405 as a candidate for the early-flowering mutation in the subsequent genetic analysis.

**TABLE 1:** Association tests between SSR marker genotypes and flowering time in the F_3_ progeny of the cross between an early-flowering mutant 1 of Nourin No. 2 (N2-ef1) and Tokachi Nagaha (TN).

**Chr**	**LG**	**SSR loci**	**Marker genotypes at SSR**				***F***	***p***	**Dorner of early-flowering allele**
			**AA**		**BB**				
			***n***	**Mean**	***n***	**Mean**			
2	D1b	Satt459	35	37.5	54	34.9	5.74	0.0187	TN
4	C1	Satt565	41	34.5	41	37.4	6.54	0.0124	N2-ef1
9	K	Satt417	42	36.7	51	34.5	4.35	0.0398	TN
9	K	Satt559	57	36.6	43	34.5	4.17	0.0438	TN
9	K	Sat_352	41	37.4	46	34.3	7.17	0.0089	TN
11	B1	Satt197	39	37.7	45	34.1	9.82	0.0024	TN
15	E	Sat_124	36	34.9	51	37.4	4.76	0.0319	N2-ef1
19	L	Sat_301	37	32.4	37	38.5	23.74	6.3E-06	N2-ef1
19	L	Sat_405	43	31.4	40	39.3	44.79	2.6E-09	N2-ef1

*One-way analysis of variance was carried out for the mean values of flowering time (days after sowing) between two homozygotes. AA and BB; homozygotes for the allele from N2-ef1 (A) and TN (B), respectively. n; number of plants tested. Chr; chromosome, LG; linkage group.*

**FIGURE 2:**
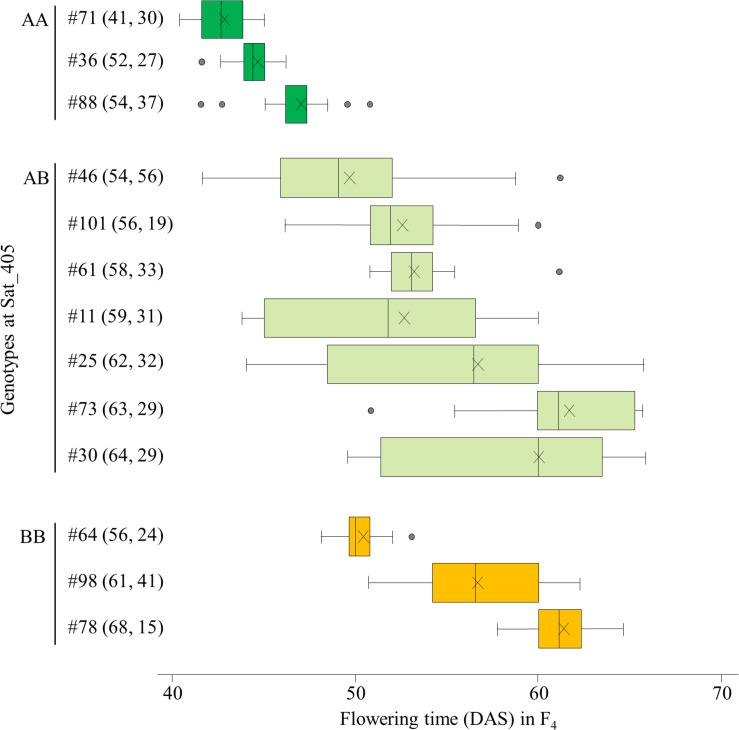
Box-plots of segregation of flowering time in the progeny of 13 F_3_ plants selected on the basis of the marker genotypes at Sat_405 in a cross between Nourin No. 2 early-flowering mutant 1 (N2-ef1) and “Tokachi Nagaha” (TN). Alleles: A, from N2-ef1; B, from “TN.” Values in parentheses represent the flowering times of the F_3_ parent (left number) and number of plants tested in the progeny (right number). The parent–offspring correlation coefficient was 0.911 (*P* < 0.001). DAS, number of days after sowing. ×, mean value.

We selected 13 F_3_ plants for the progeny test: three plants each of the AA and BB homozygotes and 7 heterozygous plants. The segregations of flowering time in their F_4_ progeny were mostly consistent with the expectation from the segregation associated with the Sat_405 genotype in the F_3_ population: plants homozygous for the A allele (#36, 71, and 88) produced mainly early-flowering progeny, whereas plants homozygous for the B allele (#64, 78, and 98) produced mainly late-flowering progeny, although the segregation patterns varied among the progeny from each homozygote. Four plants (#11, 25, 30, and 46) of the seven heterozygous plants produced progeny that segregated widely, from early-flowering plants with the AA genotype to late-flowering plants with the BB genotype (Figure 2). The parent–offspring correlation coefficient for the 13 families (*r* = 0.911) was strong and significant (*P* < 0.001).

**FIGURE 3:**
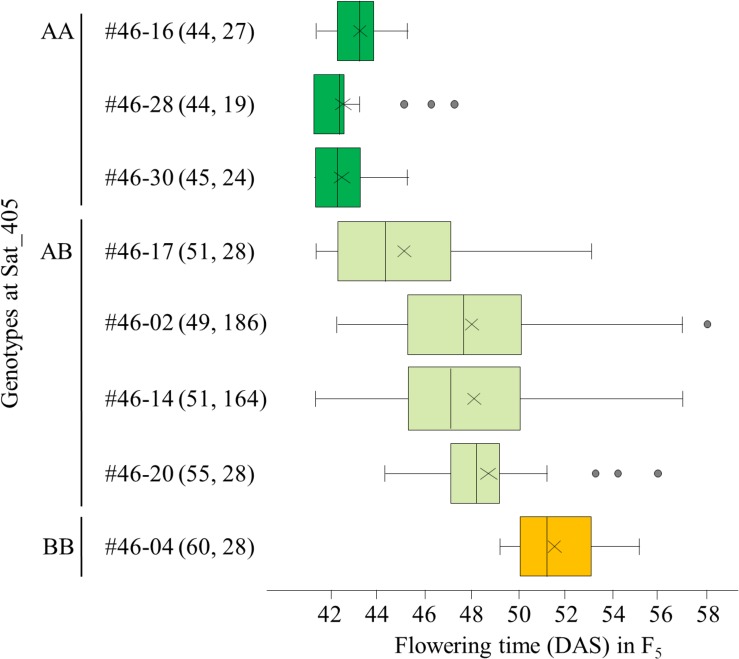
Box-plots of segregation of flowering time in the progeny of 8 F_4_ plants from family #46 of the cross between Nourin No. 2 early-flowering mutant 1 (N2-ef1) and “Tokachi Nagaha” (TN). The 8 plants were selected on the basis of the marker genotypes at Sat_405. Alleles: A, from N2-ef1; B, from “TN.” Values in parentheses represent the flowering time of the F_4_ parent (left number) and the number of plants tested in the progeny (right number). The parent–offspring correlation coefficient was 0.921 (*P* < 0.001). DAS, number of days after sowing. ×, mean value, Supplementary Figures S1, S2 show the box-plots for the other two families in this analysis.

Among the segregating families, we selected three (#11, 25, and 46) for further analyses. All three families exhibited close associations between the Sat_405 genotype of the F_4_ plants and the segregation pattern for flowering time in the progeny (Figure 3 for #46, and Supplementary Figures S1, S2 for #11 and #25, respectively), as was observed in the progeny of the F_3_ plants (Figure 2). The F_4_ plants with the AA genotype produced only early-flowering progeny in all families, whereas those with the BB genotype produced mainly late-flowering progeny, but their segregation patterns were variable, in particular in families #11 and 25 (Supplementary Figures S1, S2), suggesting that several genes in addition to the mutant gene were still segregating in these families. In contrast, in progeny of family #46, flowering time segregated mostly within a limited range of 42–58 DAS; three AA plants (#46-16, 46-28, and 46-30) and one BB plant (#46-04) produced only early-flowering and late-flowering progeny, respectively, whereas heterozygous plants (#46-2, 46-14, 46-17, and 46-20) produced progeny that segregated mostly in the range between the progeny of the AA and BB plants (Figure 3).

**FIGURE 4:**
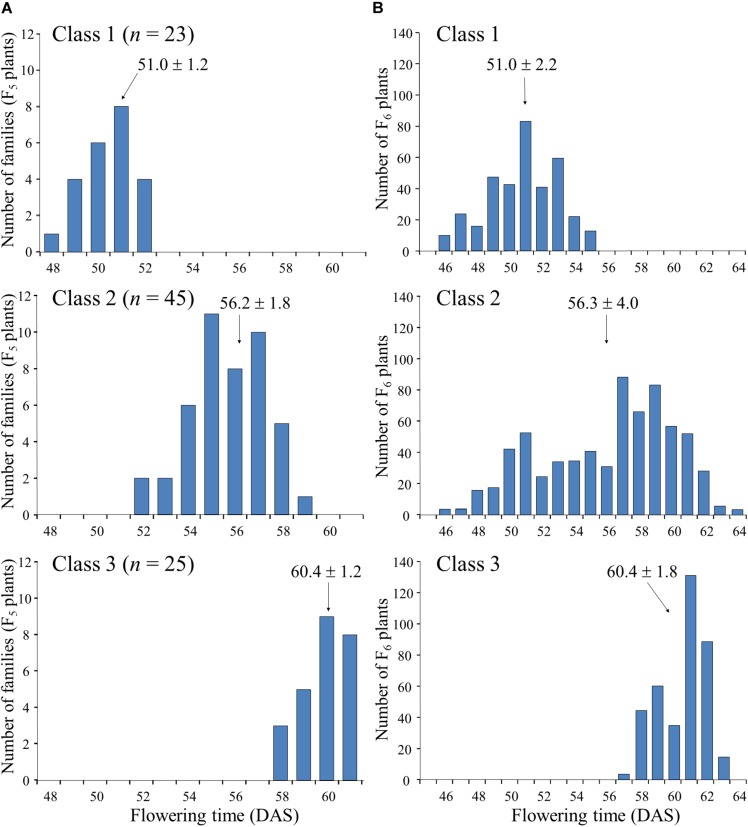
Segregation of flowering time in the progeny of F_5_ plants of a cross between Nourin No. 2 early-flowering mutant 1 (N2-ef1) and “Tokachi Nagaha” (TN). We classified 93 F_5_ plants into three classes based on the progeny test: Class 1, plants that produced only early-flowering progeny (*n* = 23); Class 2, plants whose progeny showed segregation for flowering time (*n* = 45); Class 3, plants that produced only late-flowering progeny (*n* = 25). **(A)** average flowering times of F_5_ plants, **(B)** flowering times of F_6_ progeny. DAS, number of days after sowing.

Next, we randomly selected 93 plants from the 164 F_5_ plants tested in the progeny of F_4_ plant #46-14 and tested their progeny. On the basis of the segregation pattern in the progeny, we classified the 93 plants into three classes: class 1, plants that produced only early-flowering progeny; class 2, plants that showed segregation of flowering time; and class 3, plants that produced only late-flowering progeny (Figure 4). Twenty-three plants were classified into class 1, 45 into class 2, and 25 into class 3. The segregation ratio of 23:45:25 was in good accordance with an expected 1:2:1 ratio from monogenic Mendelian inheritance (χ^2^ = 0.18, *P* = 0.91). Average flowering times in the progeny ranged from 48 to 52 DAS (with an overall average of 51.0) in the class 1 F_5_ plants and from 58 to 61 DAS (with an overall average of 60.4) in the class 3 F_5_ plants; plants in class 1 thus flowered, on average, approximately 9 days earlier than those in class 3 (Figure 4A). Average flowering times in the progeny of the class 2 F_5_ plants ranged from 52 to 59 DAS (with an overall average of 56.2), which slightly overlapped with the average flowering times of plants in classes 1 and 3. Furthermore, flowering times (46–64 DAS) of the F_6_ progeny derived from the heterozygous F_5_ plants (i.e., heterozygous progeny) overlapped the times (46–55 DAS) for the plants that were homozygous for early-flowering (class 1) and the times (57–63 DAS) for the plants that were homozygous for late-flowering (class 3); the late-flowering plants, which flowered similarly to those in class 3, segregated with higher frequency than the early-flowering plants, which flowered similarly to those in class 1 (Figure 4B). These results suggest that the segregation of flowering time in the family 46-14 was controlled mainly by a single gene, in which the early flowering from N2-ef1 was conditioned by a recessive allele (hereafter, tentatively designated *ef1*).

### Fine-Mapping of the Mutant Gene *ef1*

**FIGURE 5:**
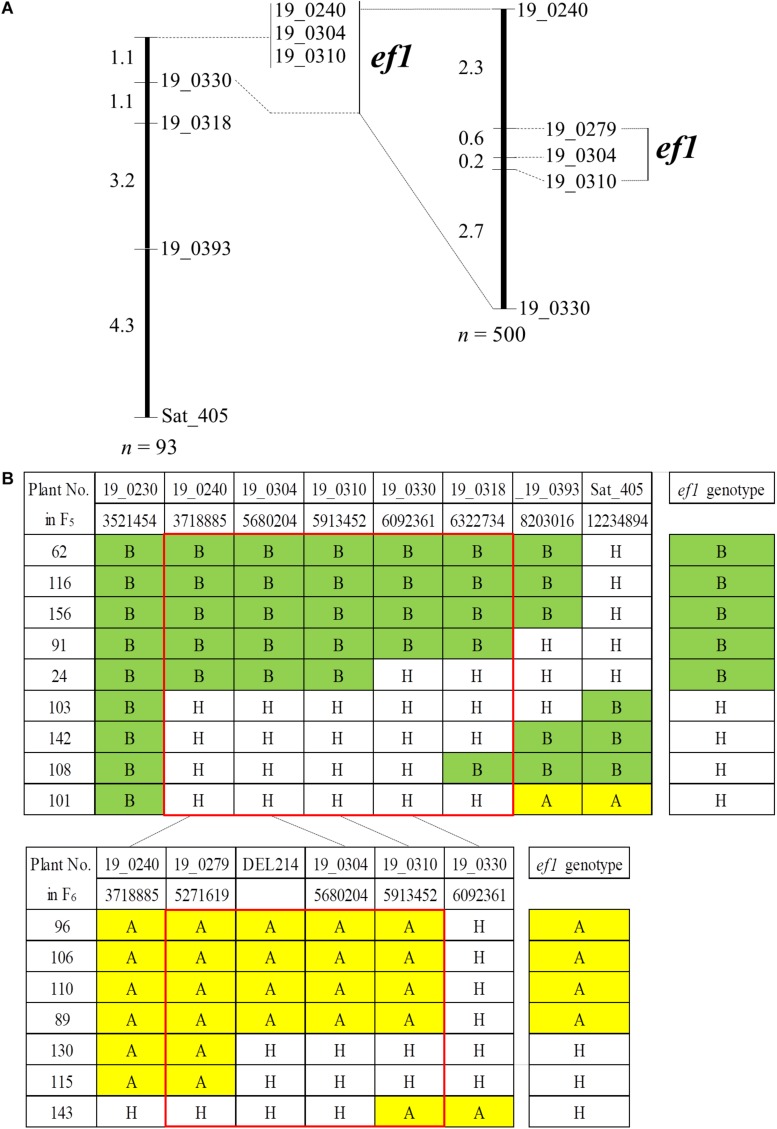
Linkage maps and graphical genotypes for the SSR markers flanking a candidate gene for early-flowering (*ef1*) from Nourin No. 2 early-flowering mutant 1. **(A)** Linkage maps for the SSR markers and *ef1*. **(B)** Graphical genotypes of recombinants among the SSR markers and association with the *ef1* genotypes estimated by the progeny test. Numbers under the SSRs are the genomic positions in the Williams 82 reference genome. Colors represent the homozygous A allele (yellow), the homozygous B allele (green), and heterozygous plants (H, white).

To delineate the genomic position of *ef1*, we analyzed genotypes for an additional six SSR markers using the 93 plants in the #46-14 family (Figure 5). All of the plants had the B allele from TN at BARCSOY_19_0230. We detected nine plants that were recombinant between BARCSOY_19_0240 and Sat_405, and constructed a linkage map of 9.7 cM (Figure 5A). The *ef1* genotypes estimated by progeny test agreed completely with the genotypes at three completely linked BARCSOY markers (BARCSOY_19_0240 to BARCSOY_19_0310) (Figure 5B).

We then analyzed an additional 500 seeds from plants heterozygous for *ef1* for BARCSOY_19_0240 and BARCSOY_19_0330. Of these, seven seeds showed recombination between the two markers, and were genotyped for three SSRs (BARCSOY_19_0279, BARCSOY_19_304, and BARCSOY_19_310). We constructed a linkage map of 5.8 cM for these five SSR markers (Figure 5A). The seven plants were then grown in the greenhouse during the winter of 2018 and the *ef1* genotypes were estimated from the segregation in the progeny in the summer of 2019. A comparison of the graphical genotypes constructed by the SSR markers and the *ef1* genotype estimated by the progeny test revealed that the *ef1* gene for early-flowering from N2-ef1 was most likely located between BARCSOY_19_0279 and BARCSOY_19_0310 (Figure 5B).

### Identification of the Molecular Basis for *ef1* Based on Resequencing Data

The region delineated by fine-mapping encompassed a 642-kbp genomic region (5.271–5.913 Mbp) of the Williams 82 genome sequence, which contained 32 annotated genes (Glyma.19G037700 to Glyma.19G040800; Supplementary Table S4). Comparison of resequencing data between N2 and N2-ef1 for the delineated genomic region in the Integrative Genomics Viewer revealed a large deletion of ∼214 kbp in N2-ef1 (Supplementary Figure S3A).

We confirmed the deletion by PCR with primers designed to sandwich it (Supplementary Figure S3A). As expected, the product amplified with primers F1 and R2 was detected only in N2-ef1, not in N2 or TN, whereas the product amplified with primers F1/R1 and F2/R2, in which R1 and F2 were designed inside the deleted region, was detected in N2 and TN, but not in N2-ef1 (Supplementary Figure S3B). Sequence analysis of the product amplified from N2-ef1 confirmed the deletion of 213,570 bp, and no insertion (Supplementary Figure S3C). By PCR using a set of three primers (F1, R1, and R2 in Supplementary Figure S3A), we determined the genotypes for the deletion for the seven recombinant plants: all plants had identical genotypes for the deletion (DEL214 marker) and BARCSOY_19_0304 (Figure 5B). Apart from the deletion, no mutation was detected to change the functions in the remaining genes. Taken together, the results indicate that the causal DNA polymorphism for the early-flowering phenotype from N2-ef1 was most likely present in the deleted 214-kbp genomic region.

The deleted region contained 11 annotated genes (Supplementary Table S4): MYB83 (Glyma.19G038100), involved in secondary cell wall biosynthesis (McCarthy et al., 2009; Zhong and Ye, 2011); NADP-dehydrogenase, which is involved in mitochondria (Glyma.19G038200); a protein in the 6-phosphogluconate dehydrogenase family (Glyma.19G038400); UDP-glucosyl transferase 85A4 (Glyma.19G038500); an ATPase E1-E2 type family protein/haloacid dehalogenase-like hydorolase family protein (Glyma.19G038600); glutamine dumper 3 (Glyma.19G038700); an ortholog of *Arabidopsis CONSTANS* (Glyma.19G039000; COL2b); NSP-interacting kinase 1 (Glyma.19G039100); and three proteins with uncharacterized functions (Glyma.19G038300, Glyma.19G038800, and Glyma.19G038900). A survey of the Flowering Interactive Database (FLOR-ID)^6^ storing 306 *Arabidopsis* flowering genes (Bouché et al., 2016) indicated that no annotated genes homologous to *Arabidopsis* flowering genes existed in the deleted region except for Glyma.19G039000. The survey further showed that out of the 21 annotated genes in the flanking regions of deletion, Glyma.19G040300 was homologous to a MYB-RELATED PROTEIN 1 (AT5G18240) involved in photoperiod pathway of flowering. However, Glyma.19G040300 was a dysfunctional gene encoding a truncated protein of 93 amino acids, despite that its homoeolog, Glyma.02G070900, and AT5G18240 had 416 and 402 amino acids, respectively. The resequencing data indicated that N2 and N2-ef1 possessed the same dysfunctional gene as Williams 82. The micro-syntenic relationships of the 11 genes in the deletion with their homoeologous copies depicted with reference to the Williams 82 genome sequence shows no distinct micro-synteny between the deleted region and the other genomic regions, but all of the genes except for the mitochondrial gene had at least one homolog in different regions of seven chromosomes (Supplementary Figure S4).

### Effects of *ef1* on Flowering

To characterize the effects of *ef1* on flowering, we compared flowering time under different photo-thermal conditions between NILs for the *ef1* and wild-type alleles. We selected two heterozygous plants (#46-14-32 and #46-14-69) from the F_5_ progeny of #46-14. On the basis of the marker genotype at BARCSOY_19_0304, two F_5__:__6_ plants – one early-flowering with the AA genotype and the other late-flowering with the BB genotype – were randomly selected from each progeny and were used as two sets (#46-14-32 and #46-14-69) of NILs for the *ef1* and wild-type alleles, respectively.

**FIGURE 6:**
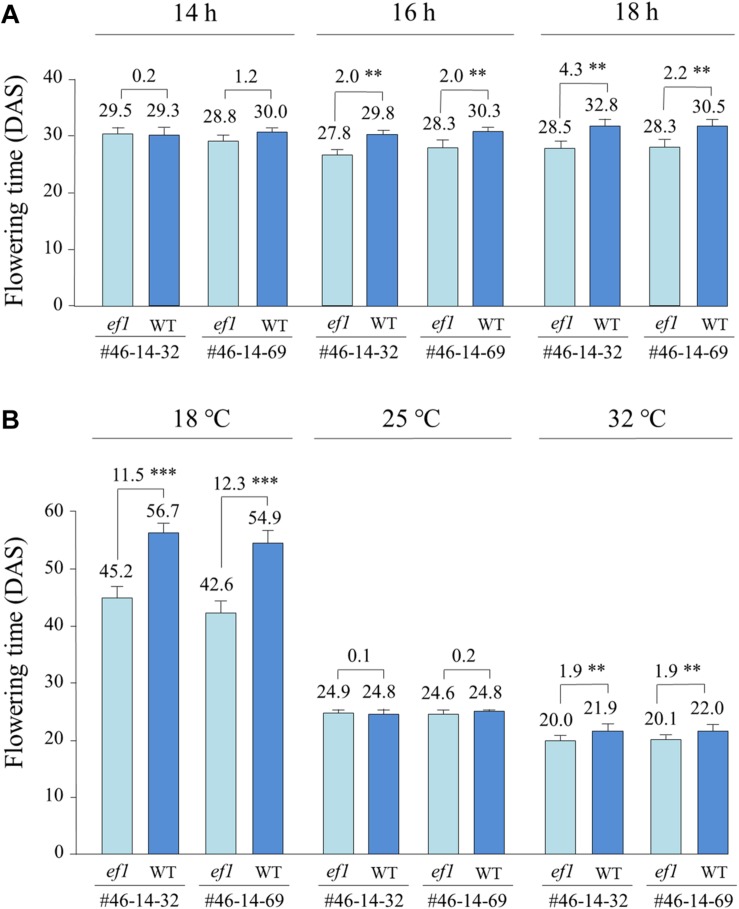
Differences in flowering time between two sets of NILs (#46-14-32 and #46-14-69) with the *ef1* and wild-type (WT) alleles under different photo-thermal conditions: **(A)** three photoperiods under a constant ambient temperature of 25°C in the greenhouse, **(B)** a constant daylength of 16 h under three ambient temperatures in the growth chambers. Differences in average flowering time between NILs are presented above the bars. ^∗∗^*P* < 0.01; ^∗∗∗^*P* < 0.001. DAS, number of days after sowing.

Under a constant ambient temperature of 25°C, the *ef1* lines flowered at almost the same time as the wild-type lines under a 14 h daylength (with no significant difference), but flowered, on average, 2.0 days earlier than the latter under 16 h and 2.2–4.3 days earlier under 18 h (Figure 6A). The different alleles also conferred different responses to changes of ambient temperature (Figure 6B). At 25°C under a 16 h daylength, flowering time was almost the same between the NILs (with no significant difference). At 32°C, flowering was promoted slightly in all lines; the *ef1* lines flowered 1.9 days earlier than the wild-type lines (Figure 6B). In contrast, at 18°C, the differences in flowering time between NILs were enlarged to 11.5 and 12.3 days in #46-14-32 and #46-14-69, respectively. The reduction of ambient temperature from 25 to 18°C greatly delayed flowering in all lines, but the effect differed between the NILs: by 18–20 days in the *ef1* lines and by 30–32 days in the wild-type lines (Figure 6B).

**FIGURE 7:**
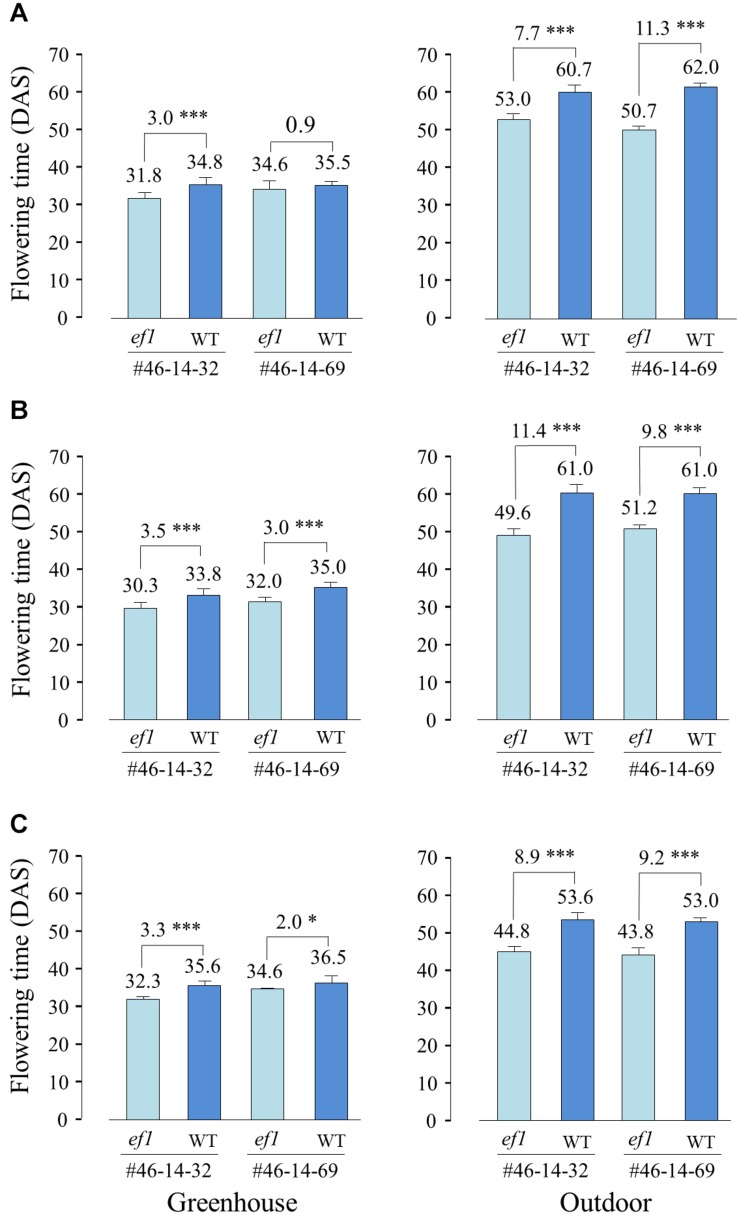
Differences in flowering time between two sets of NILs (#46-14-32 and #46-14-69) with the *ef1* and wild-type (WT) alleles in the greenhouse and outdoors under natural daylengths at three different sowing times: **(A)** first sowing (11 May), **(B)** second sowing (21 May), and **(C)** third sowing (31 May). Differences in average flowering time between NILs are presented above the bars. ^∗^*P* < 0.05; ^∗∗∗^*P* < 0.001;

We also compared the flowering times of NILs sown at different dates between the greenhouse and outdoor conditions. Average daylengths at 20 DAS were almost the same among the three sowing dates (ca. 15 h 10 min to 15 h 20 min). However, average temperatures during the same periods were 5–6°C lower outdoors than in the greenhouse. In the greenhouse, the *ef1* lines flowered, on average, 0.9–3.5 days earlier than the wild-type lines, whereas they flowered 7.7–11.4 days earlier outdoor (Figure 7). The low temperatures outdoors delayed flowering by, on average, 27, 26, and 17 days at the 1st, 2nd, and 3rd sowing, respectively in the wild-type lines and by 18, 19, and 11 days in the *ef1* lines. The inhibition of flowering by lower temperatures was thus reduced in plants with the *ef1* allele.

### Expression Profiles of Flowering Genes Under Different Thermal Conditions

**FIGURE 8:**
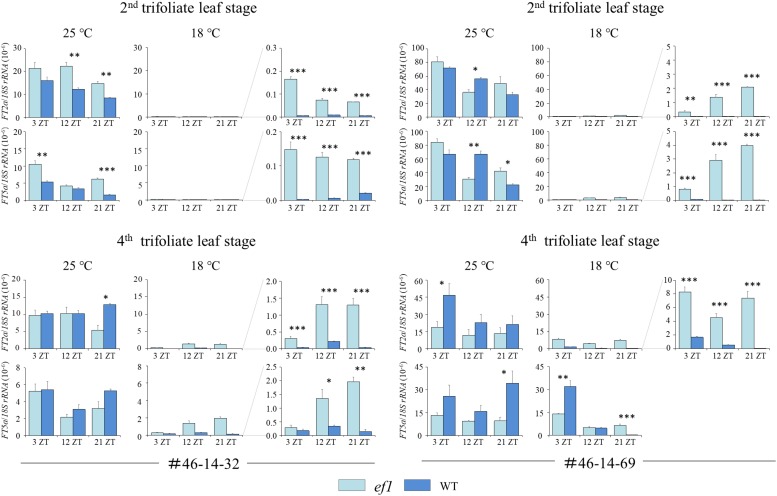
Transcript levels of *FT2a* and *FT5a* at the second and fourth trifoliate leaf stages in two sets of NILs (#46-14-32 and #46-14-69) with the early-flowering (*ef1*) and wild-type (WT) alleles at 18 and 25°C. The enlarged view for the expression levels at 18°C is presented in the right column. ZT values are Zeitgeber times. Error bars are technical repeats (*n* = 3). ^∗^*P* < 0.05; ^∗∗^*P* < 0.01; ^∗∗∗^*P* < 0.001.

We analyzed transcript abundances of *FT2a* and *FT5a* at the second and fourth trifoliate leaf stages at 18 and 25°C (Figure 8). At 25°C, there were no consistent differences in *FT2a* or *FT5a* transcripts between the *ef1* and wild-type alleles at either growth stage. However, the expression of both genes was strongly decreased by low temperature at both stages in *FT2a* and at the second trifoliate-leaf stage in *FT5a*. The decrease caused by low temperature was greater at the second trifoliate leaf stage; the transcript levels at 18°C decreased to approximately 5 and 1%, respectively, of the values at 25°C in #46-14-69 and #46-14-32. Despite the strong repression, the transcript levels at 18°C were significantly higher in the *ef1* lines than the wild-type lines at any time points. At the fourth trifoliate leaf stage, the decrease was smaller, particularly in the *ef1* lines: the *FT2a* expression decreased to 10% of the level at 25°C in the #46-14-32 NILs and to 50% of the level at 25°C in the #46-14-69 NILs, and the *FT5a* expression decreased to only 50% of the level at 25°C in the #46-14-32 NILs, versus no decrease in the #46-14-69 NILs. Similarly as in the second trifoliate leaf stage, the transcript levels were higher in the *ef1* lines than the wild-type lines at 18°C, except for *FT5a* in the 46-14-69 NILs.

**FIGURE 9:**
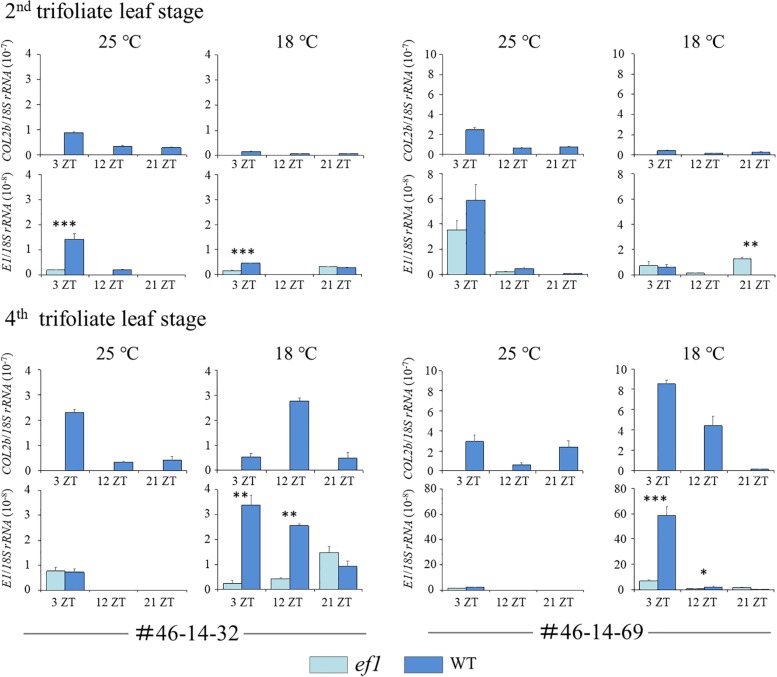
Transcript levels of *COL2b* and *E1* at the second and fourth trifoliate leaf stages in two sets of NILs (#46-14-32 and #46-14-69) with the early-flowering (*ef1*) and wild-type (WT) alleles at 18 and 25°C. ZT values are Zeitgeber times. Error bars are technical repeats (*n* = 3). ^∗^*P* < 0.05; ^∗∗^*P* < 0.01; ^∗∗∗^*P* < 0.001.

We also analyzed transcript levels for *COL2b* and *E1* (the repressor for both *FT2a* and *FT5a*) (Figure 9). At the second trifoliate leaf stage, the transcript levels for *COL2b* and *E1* mostly decreased in response to decreasing temperature. There was no constant association between *COL2b* or *E1* transcripts and the two *FT* genes (Figures 8, 9). In contrast, at the fourth trifoliate leaf stage, low temperature upregulated *COL2b* expression in the #46-14-69 NIL with the wild-type allele and shifted the expression peak from 3 ZT at 25°C to 12 ZT at 18°C in the #46-14-32 NIL. Intriguingly, *E1* expression was also upregulated only in the wild-type line among the two sets of NILs; the effect of cool temperature was remarkable in the #46-14-69 NIL.

## Discussion

### Early Flowering of the *ef1* Mutant Can Be Attributed to a Deletion in Chromosome 19

We determined that the causal DNA polymorphism for the early flowering *ef1* mutant generated by ion-beam radiation was a 214-kbp deletion in the upper part of chromosome 19 (from positions 5,327,094 to 5,540,664 in the Williams 82 reference genome). We crossed the mutant line N2-ef1 with TN, not with the parental line N2, to facilitate marker-assisted analysis for mapping the mutant gene. Both TN and N2 had the same maturity genotype alleles at *E1*–*E4* (*E1*/*e2*/*e3*/*E4*), but TN flowered 20 days earlier than N2 in Hokkaido. Consequently, a number of genes for flowering, together with the *ef1* mutation, were segregated in the progeny, but the *ef1* mutation appears to have had the largest promotive effect on flowering.

A number of mutant lines with altered flowering have been selected from the same mutant population, but most of these lines had yellowish leaves with reduced chlorophyll contents (Mikuriya et al., 2017). The mutant N2-ef1 and its derived lines, however, had no phenotypic aberration in plant architecture or growth, even though 11 genes were deleted. Soybean is a paleopolyploid species, which has multiple gene copies as a result of two rounds of polyploidy (Schmutz et al., 2010). The deleted region in N2-ef1 has complex microsyntenic relationships with genomic regions of other chromosomes. However, 10 of the genes (except for a mitochondrial gene) have homoeologous copies scattered among seven chromosomes, which are probably responsible for tolerance of the large deletion in N2-ef1 (Supplementary Figure S3).

The annotated genes in the deleted region include *COL2b*, a soybean ortholog of *Arabidopsis CONSTANS*. The other genes encode proteins involved in cell metabolic pathways, such as the pentose phosphate pathway, glycosylation, ATP biosynthesis, amino acid export, and the mitochondrial electron transport chain, none of which has been implicated in flowering time control in *Arabidopsis* and the other plant species. *COL2b* may therefore be the best candidate for the early flowering of the mutant lines. Since no QTL for time to flowering has been detected so far in the genomic region flanking the deletion, the early-flowering mutation in N2-ef1 appears to be a novel genetic resource for improving our understanding of the molecular mechanisms of flowering and to support development of new cultivars in soybean.

### The Mutation Mitigates the Repression of Flowering by Cool Temperatures

Comparison of flowering times between NILs for the mutant *ef1* and wild-type alleles under different photo-thermal conditions revealed that the *ef1* allele has a unique characteristic: it is involved in the thermal regulation of flowering, and particularly in the response to cool temperatures (Figure 6). The NILs with the *ef1* and wild-type alleles flowered at almost the same time at 25°C with a 16 h daylength, but responded differently when the temperature was reduced to 18°C: the flowering was markedly delayed in NILs with the wild-type allele, but the effect was mitigated in NILs with the *ef1* allele (Figure 6B). Similar results were obtained in the greenhouse and outdoor experiments, in which the ambient temperature averaged 5–6°C lower outdoors for all sowing dates; outdoors the NILs with the *ef1* allele flowered 8–11 days earlier than those that carried the wild-type allele, but less than 3 days earlier in the greenhouse (Figure 7). Increasing the ambient temperature from 25 to 32°C also influenced flowering times differentially between the NILs, but the effect was smaller than the response to low temperatures (Figure 6B). Therefore, the mutation appears to mainly moderate the floral repression induced by cool temperatures.

The different flowering times at cool temperatures reflected different levels of *FT2a* and *FT5a* transcripts between the NILs (Figure 8). Cooler temperatures greatly decreased the expression of *FT2a* and *FT5a* at both growth stages. However, the repression was moderated in the NIL with the *ef1* allele. In contrast, we observed no large and consistent difference between the NILs in the expression levels of *FT2a* and *FT5a* at 25°C. The effect of the early-flowering *ef1* mutation may therefore be attributed to its alleviation of the repression of *FT2a* and *FT5a* expression at cool temperatures.

### *COL2b* Is a Candidate for Flowering Repression by Cool Temperatures

COL2b is one of four CO homologs, two sets of homoeologous copies (COL1a/COL1b and COL2a/COL2b), with high amino acid similarity to *Arabidopsis* CO (Wu et al., 2014). The four CO homologs belong to a single clade connected to *Arabidopsis* CO, COL1, and COL2; they are more similar to COL2 out of the three *Arabidopsis* CO/COL proteins, whereas CO is closer to COL2a/COL2b than COL1a/COL1b (Wu et al., 2014). When ectopically expressed under the control of the cauliflower mosaic virus 35S promoter, each of the four homologs fully complements the late flowering of the *co-1* mutant in *Arabidopsis*, suggesting that they retain the function of *CO* and are possible floral inducers in soybean (Wu et al., 2014). However, analyses of overexpression and artificially induced missense mutants revealed that both *COL1a* and *COL1b* inhibit flowering under long-day conditions (Cao et al., 2015), as in the case of *HEADING DATE 1* (*Hd1*), a rice ortholog of *CO* (Yano et al., 2000). Unlike *Hd1*, the overexpression of *COL1a* did not promote flowering in short-day conditions, although the *FT2a* and *FT5a* expressions were slightly upregulated (Cao et al., 2015). The functions of *COL2a* and *COL2b* in soybean flowering have not yet been determined.

*CO* is a key integrator in the photoperiod pathway of *Arabidopsis* (Song, 2016). The expression of *CO* is upregulated in late afternoon under long-day conditions by the degradation of CYCLING DOF FACTOR family members, which function as repressors of *CO* transcription, by the FLAVIN-BINDING, KELCH REPEAT, F-BOX 1 (FKF1)–GI complex, and the accumulated CO protein is stabilized by GI, FKF1, and PHYA to induce *FT* expression (Song, 2016). *CO* is also involved in the thermal regulation of flowering, particularly in response to high temperatures. Increasing the ambient temperature to 27°C accelerates flowering even under non-inductive short-day conditions (Balasubramanian et al., 2006; Sanchez-Bermejo et al., 2015). This acceleration of flowering by high temperature is mediated by CO and by a basic helix-loop-helix transcription factor, PHYTOCHROME INTERACTING FACTOR 4 (PIF4), which functions as an activator for *FT* expression; CO forms a protein complex with PIF4 to activate *FT* expression under high-temperature short-day conditions (Fernández et al., 2016).

A reduction in ambient temperature from 22 to 16°C delays flowering in *Arabidopsis* (Strasser et al., 2009). This process involves a number of molecular mechanisms: (1) the occupation of histone H2A variant H2A.Z nucleosomes on the *FT* locus to inhibit the binding of the *PIF4* transcription factor to the promoter, (2) destabilization of the CO protein by HIGH EXPRESSION OF OSMOTICALLY RESPONSIVE GENES 1, an E3 ubiquitin ligase, and (3) repression of floral integrators (*FT*, *TWINSISTER OF FT*, and *SUPPRESSOR OF CONSTANS OVEREXPRESSION 1*) by repressor complexes of SHORT VEGETATIVE PHASE with functional transcripts of *FLOWERING LOCUS M* (*FLM-*β) and *MADS AFFECTING FLOWERING 2* (*MAF2ver1*) (Ratcliffe et al., 2003; Strasser et al., 2009; Jung et al., 2012; Gu et al., 2013; Lee et al., 2013; Posé et al., 2013; McClung et al., 2016; Song, 2016). The evening complex, which consists of ELF3, ELF4, and LUX ARRHYTHMO (LUX), represses its direct targets, *PSEUDO RESPONSE REGULATOR* 7 (*PRR7*), *GI*, and *LUX*, and delays flowering under cool temperatures (Mizuno et al., 2014). The loss of function of *ELF3* mitigates the repression of flowering by cool temperatures to accelerate flowering under these conditions (Strasser et al., 2009). TERMINAL FLOWER 1 (TFL1) also functions in the control of flowering as a thermal sensor; ELF3 and TFL1 confer the thermal regulation of flowering through different interactions with the PHYTOCHROME B and CRYPTOCHROME photoreceptors (Strasser et al., 2009).

The thermal regulation of flowering in response to ambient temperatures has also been investigated in rice and barley. In rice, the reduction of ambient temperature from 27 to 23°C delays flowering by downregulating expression of *Hd3a* and *RFT1* (rice *FT* orthologs) and of *Ehd1* (an inducer for *Hd3a* and *RFT1*) (Luan et al., 2009; Song et al., 2012). This repression is caused by increased transcript levels of *Ghd7*, a rice-specific gene that encodes a CCT domain protein, at low temperature, particularly under long-day conditions (Song et al., 2012). This suggests that *Ghd7* plays a crucial role in controlling flowering in response to photoperiod and ambient temperature (Song et al., 2012). *Hd1*, a rice ortholog of *CO*, is essential for the induction of *Ghd7* expression under long-day conditions (Song et al., 2012). *Hd1* may therefore be involved in thermal regulation of flowering through the regulation of *Ghd7*. In barley (*Hordeum vulgare*), high ambient temperature (28/24°C, day/night) retards the reproductive development of spring barley that carries dysfunctional alleles of both *PHOTOPERIOD1* (*Ppd*-*H1*, the barley homolog of *Arabidopsis PRR*) and *VRN-H2* (the barley homolog of rice *Ghd7*), relative to the control temperature (20/16°C, day/night) (Ejaz and von Korff, 2017). Both the functional *Ppd-H1* allele and the loss-of-function allele of *HvELF3*, an upstream regulator of *Ppd-H1*, which represses *Ppd-H1* expression at night (Faure et al., 2012), accelerate flowering and floral development under high temperatures (Ejaz and von Korff, 2017). Two homologs of *Arabidopsis CO*, *HvCO1*, and *HvCO2*, upregulate *VRN-H2*, resulting in reduced expression of *HvFT1* (the barley homolog of *FT*), and this delays flowering under both long-day and short-day conditions when it is ectopically overexpressed in the winter genotype that carries a functional *VRN-H2* allele; however, in the spring genotype that carries a dysfunctional *vrn-H2* allele, *HvCOL2* can function as an activator of flowering, depending on the *Ppd-H1* genotype (Mulki and von Korff, 2016). The findings obtained in these plant species suggest that stimuli from photoperiod and ambient temperature are integrated by key genes that control the flowering time.

We found that expression of *FT2a* and *FT5a* was strongly downregulated by cool temperatures at both growth stages. However, their effects differed between the NILs. The expression of *FT2a* and *FT5a* in the *ef1* lines that lacked *COL2b* was low but detectable and promoted flowering even at 18°C, but was strongly repressed in the wild-type lines (Figure 8). It is therefore tempting to hypothesize that *COL2b* functions as a floral repressor in response to cool temperature, as in the case of *COL1a* and *COL1b*, which are involved in the inhibition of flowering under long-day conditions (Cao et al., 2015). In the wild-type lines, the cool temperature upregulated the expression of *COL2b* or shifted the peak from 3 ZT to 12 ZT at the fourth trifoliate leaf stage, but the expressions of *COL2b* were slightly decreased in response to cool temperature at the second trifoliate leaf stage; no consistent correlations were detected between the expression levels of *COL2b* and *FT2a* or *FT5a* (Figures 8, 9). COL2b may therefore repress *FT2a* and *FT5a* expressions especially in response to cool temperature, irrespectively of its transcript abundances; in the *ef1* lines, the lack of COL2b may moderate the repression of flowering by cool temperatures.

Intriguingly, cold temperatures also highly upregulated the expression of *E1* in the wild-type lines at the fourth trifoliate leaf stage, but had no effect in the NILs with the *ef1* allele (Figure 9). *E1* plays a central role as a repressor for *FT2a* and *FT5a* in the control of flowering under long days (Xia et al., 2012; Xu et al., 2015), but it also inhibits flowering under short-day conditions in plants that carry the loss-of-function allele at the *J* locus that encodes EFL3, which inhibits *E1* expression under short-day conditions by directly binding to the promoter (Lu et al., 2017). *COL2b* might therefore control the expression of *FT2a* and *FT5a* directly or indirectly through upregulation of *E1* expression under cool temperatures. Additional research will be required to determine whether ectopically expressed functional *COL2b* restores the floral repression by cool temperature in the NILs that carry the *ef1* allele, as was observed in plants with the wild-type allele, by inhibiting *FT2a* and *FT5a* expression, and whether this induces *E1* expression.

## Concluding Remarks

Despite their effects on reproductive growth, the molecular and genetic mechanisms involved in thermal regulation of flowering and maturation are not fully understood in soybean. The variation in flowering time among soybean cultivars is attributed mainly to variations at the loci involved in photoperiod sensitivity and to variations in the floral integrators *FT2a* and *FT5a*. Diverse genetic variation at these loci makes it complicated to characterize the genetic variation underlying differences in the thermal responses among cultivars. The different responses of flowering to cool temperature between the NILs with the *ef1* and those with the wild-type alleles that we observed in this study may indicate that the temperature-dependent response of flowering is not solely a product of promotive or suppressive effects on plant growth by temperature. Rather, the genes underlying photoperiod sensitivity, such as *CO* orthologs and *E1*, might be the key players in thermal regulation of flowering in soybean, as is the case in key genes such as *Ghd7*/*VRN2-H2* and *Hd1*/*HvCO1*/*2* that have been reported in rice and barley. The early-flowering mutation characterized in this study will be a useful genetic resource both to improve our understanding of the molecular mechanisms that underlie thermal regulation of flowering and to support the development of new cultivars that are adapted to cool environments, which often create cold stress in soybean.

## Data Availability Statement

The sequencing data used in this study has been submitted to NCBI under BioProject accession number PRJNA600284.

## Author Contributions

JA, AK, and YH selected the mutant. JZ, TS, MI, SW, and TY conducted genetic analyses and fine-mapping. JZ, MD, and BL conducted sequencing analyses. JZ and JA evaluated flowering of NILs. JZ, MX, and TY conducted the expression analyses. ZJ and JA drafted the manuscript with edits from MX, SW, TY, TS, MI, YH, and AK. All authors read and approved the final manuscript.

## Conflict of Interest

The authors declare that the research was conducted in the absence of any commercial or financial relationships that could be construed as a potential conflict of interest.

## Footnotes

1 https://phytozome.jgi.doe.gov/pz/portal.html

2 https://bioweb.pasteur.fr/packages/pack@bowtie2@2.2.9

3 http://broadinstitute.github.io/picard

4 https://gatkforums.broadinstitute.org/gatk/

5 https://software.broadinstitute.org/software/igv/

6 http://www.phytosystems.ulg.ac.be/florid/
