# Supplementary figures and images for: A Soybean Deletion Mutant That Moderates the Repression of Flowering by Cool Temperatures
Source: Front Plant Sci. 2020 Apr 15;11:429. doi: 10.3389/fpls.2020.00429 (PMC7175460; doi:10.3389/fpls.2020.00429)

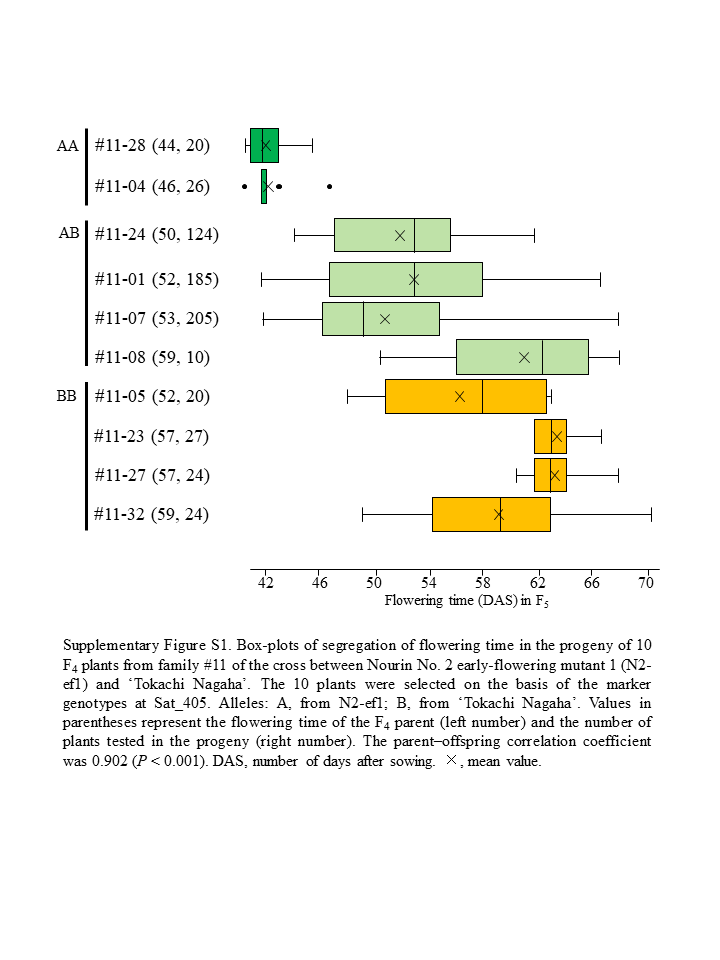

Supplement: Supplementary file 2 [file Image_1.tif]

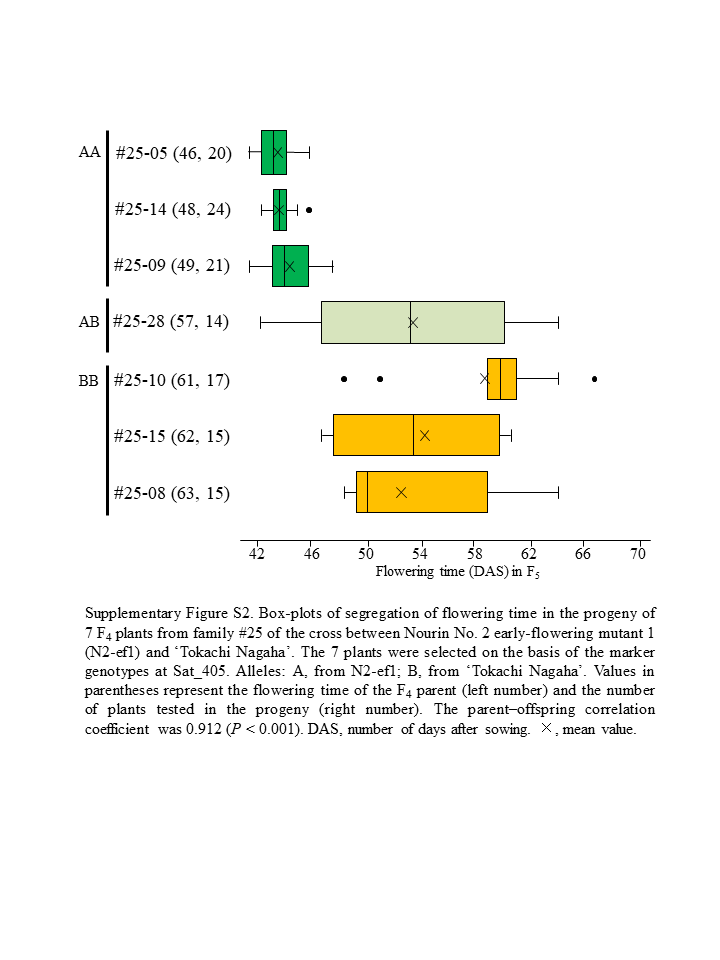

Supplement: Supplementary file 3 [file Image_2.tif]

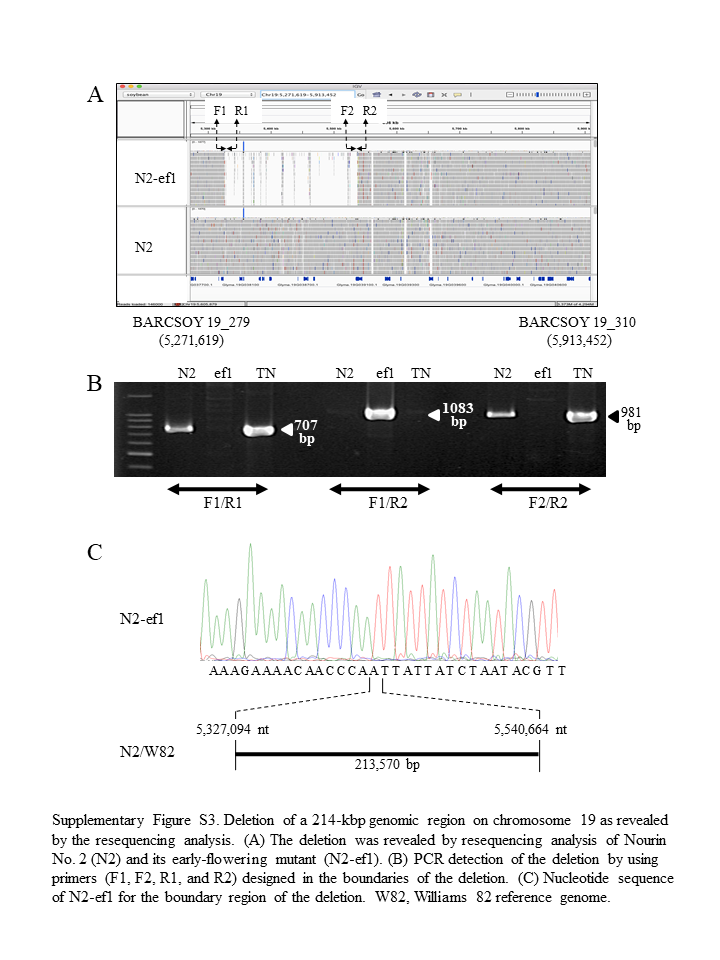

Supplement: Supplementary file 4 [file Image_3.tif]

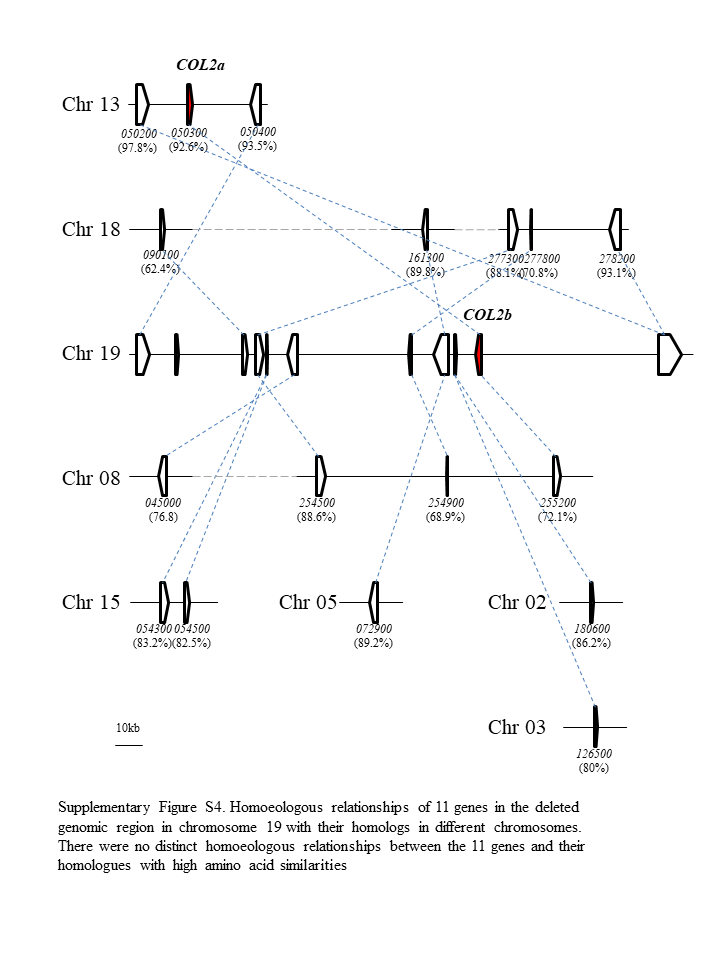

Supplement: Supplementary file 5 [file Image_4.tif]
